# Threading Granules in Freiburg. 2^nd^ International Symposium on “One Mitochondrion, Many Diseases - Biological and Molecular Perspectives”, a FRIAS Junior Researcher Conference, Freiburg im Breisgau, Germany, March 9^th^/10^th^, 2016.

**DOI:** 10.15698/mic2016.11.540

**Published:** 2016-11-04

**Authors:** Ralf J. Braun, Ralf M. Zerbes, Florian Steinberg, Denis Gris, Verónica I. Dumit

**Affiliations:** 1 Institute of Cell Biology, University of Bayreuth, 95440 Bayreuth, Germany.; 2 Institute for Biochemistry and Molecular Biology, University of Freiburg, 79104 Freiburg, Germany.; 3 ZBSA Center for Biological Systems Analysis, AG Steinberg, University of Freiburg, 79104 Freiburg, Germany.; 4 Program of Immunology, Department of Pediatrics, CR-CHUS, Faculty of Medicine and Health Sciences, University of Sherbrooke, Sherbrooke, QC, Canada.; 5 ZBSA Center for Biological Systems Analysis, Core Facility Proteomics, University of Freiburg, 79104 Freiburg, Germany.

**Keywords:** mitochondria, neurodegeneration, cancer, mitochondrial disorders, mitochondrial dynamics, mitochondrial protein homeostasis, mitophagy, mitochondrial inner membrane structure, oxidative stress, apoptosis, necrosis, inflammation, Saccharomyces cerevisiae

## Abstract

Altered mitochondrial activities play an important role in many different human disorders, including cancer and neurodegeneration. At the Freiburg Institute of Advanced Studies (FRIAS) Junior Researcher Conference “One Mitochondrion, Many Diseases - Biological and Molecular Perspectives” (University of Freiburg, Freiburg, Germany), junior and experienced researches discussed common and distinct mechanisms of mitochondrial contributions to various human disorders.

## INTRODUCTION

Mitochondria (greek: μίτος & χονδρίον, mitos & chondrion, i.e., thread & granule) are the power houses of eukaryotic cells, and are pivotally involved in essential metabolic processes, including iron/sulfur cluster and heme biosynthesis. Mitochondria are highly dynamic organelles that constantly fuse (resulting in thread-like structures) and divide (forming granular structures). They move along the cytoskeleton, and surplus or severely damaged organelles are degraded. The degradation occurs via mitophagy, i.e., a selective form of autophagy, where a double-membrane completely encloses the organelles. The resulting mitochondria-containing autophagsomes finally fuse with the lysosomes or vacuoles, where the degradation takes place with the help of lysosomal or vacuolar proteases. The internal structure of mitochondria highly varies depending on the demands of the cells. Critical damage of mitochondria or altered mitochondrion-associated processes are linked to many human disorders, including neurodegeneration, cancer, and aberrant inflammatory processes. On March 9^th^/10^th^, when spring was approaching, 100 scientists from Freiburg (Germany), as well as from Europe, and from overseas attended the 2^nd^ International Symposium “One mitochondrion, many diseases”. Due to the generous support of the Freiburg Institute of Advanced Studies (FRIAS) of the University of Freiburg (Germany), the researchers presented their recent data on physiological and pathophysiological processes involving mitochondria and their relevance for cellular homeostasis and cellular dysfunctions underlying various human disorders.

## DEREGULATED MITOCHONDRIAL PROTEIN HOMEO-STASIS AND ITS ROLE IN DISEASE

**Jörn Dengjel** (University of Fribourg, Fribourg, Switzerland) gave the opening lecture to the symposium. He introduced how mitochondrial dysfunction is linked to various human disorders, including mitochondrial disorders and myopathies, as well as complex disorders such as neurodegenerative disorders. He focused his talk on mitochondrial homeostasis by mitophagy, which has been proposed to be critical especially for Parkinson’s disease. In his previous work dissecting mitophagy in baker’s yeast using a quantitative proteomic approach, he elucidated that distinct mitochondrial matrix proteins are sorted into mitochondrial entities which are then degraded via mitophagy 1,2,3. These hitherto unknown sorting mechanisms prior to mitophagy determine mitochondrial protein homeostasis, and potentially could play important roles in modulating mitochondrial (dys)function in health and disease.

**Jan Riemer** (University of Cologne, Cologne, Germany) talked about the oxidation of thiols in mitochondrial respiratory chain assembly and calcium signaling. He introduced into the mechanisms of the oxidative folding machinery. He focused his talk on how this machinery can work in a reducing environment, and how a crucial disulfide bond regulates Ca^2+^ signaling 4,5.

The majority of mitochondrial proteins is synthesized as precursor proteins in the cytosol and then imported into mitochondria. In most cases, the precursor proteins comprise N-terminal presequences, which are cleaved after import by mitochondrial presequence proteases. **Nora Vögtle** (University of Freiburg, Freiburg, Germany) described the physiological role of these mitochondrial presequence proteases and their impact on mitochondrion-modulated disorders 6,7. The activities of these proteases are tightly regulated by feedback loops and pathological proteins, such as the Alzheimer’s disease-associated peptide β-amyloid, which impairs turnover of presequence peptides with detrimental consequences 8.

**Ralf Braun** (University of Bayreuth, Bayreuth, Germany) demonstrated that accumulation of mutant Alzheimer’s disease-associated ubiquitin impairs the ubiquitin-proteasome system (UPS), leads to the aberrant enrichment of enzymes in mitochondria, which elicit mitochondrial dysfunction and cell death 9,10. Intriguingly, promoting the mitochondrion-associated branch of the UPS reduced the cellular levels of these enzymes and protected mitochondria and cells from the detrimental effects of mutant ubiquitin. These data indicate a pivotal role of UPS (dys)function in controlling metabolic activities in mitochondria with a potential relevance for human diseases.

**Julia Ring** (University of Graz, Graz, Austria) described a yeast model expressing the Alzheimer’s disease-associated hydrophobic peptide β-amyloid. She demonstrated that β-amyloid localizes to mitochondria executing oxidative stress and cell death. She identified factors that modulate the aberrant accumulation of these detrimental peptides at the mitochondrial outer membrane.

## FUNCTIONAL ARCHITECTURE AND DYNAMICS OF MITOCHONDRIA

The inner-mitochondrial structure is highly dynamic and adapts to the needs of the cell. The components and mechanisms shaping inner mitochondrial membranes are currently elucidated. **Martin van der Laan** (Saarland University, Homburg, Germany) identified the mitochondrial contact sites and cristae-organizing system (MICOS), which controls mitochondrial inner membrane morphology, and enables multifunctional organization of mitochondria 11. He focused his talk on the role of Mic10, which is the main component of the MICOS backbone in baker’s yeast 12,13. He presented a hypothetical model how Mic10 shapes the mitochondrial cristae. **Ralf Zerbes** (University of Freiburg, Freiburg, Germany) delineated the role of another MICOS component. He described that Mic60 and its interacting partner Mib26 link respiratory chain assembly with cristae formation. He proposed that MICOS is needed to provide the right localization of distinct respiratory chain components and assembly intermediates, i.e., the mitochondrial ultrastructure determines mitochondrial function.

Mature mRNA contain unconventional open reading frames (AltORFs) located in the untranslated regions or overlapping the reference ORFs (RefORFs) in non-canonical +2 and +3 reading frames 14. **Xavier Roucou** (University of Sherbrooke, Sherbrooke, QC, Canada) identified AltMID51 encoded by an AltORF in the mRNA encoding the RefORF of the mitochondrial dynamics protein of 51 kDa (MID51). AltMID51 homodimerized in mitochondrial foci. The ORFs encoding AltMID51 and MID51 are evolutionary tightly associated, and overexpression of both proteins triggered mitochondrial fragmentation. These and other data propose that AltMID51 and MID51 are both involved in mitochondrial fragmentation.

**Denis Gris** (University of Sherbrooke, Sherbrooke, QC, Canada) analyzes the role of proteins involved in the innate immune response on the survival of neurons. He is working on NLRX1, the only mitochondrial member of the nucleotide-binding domain leucine-rich repeat-containing protein (NLR) family. He proposed that NLRX1 could be a molecular switch controlling both neuronal survival and inflammatory signaling 15. NLRX1 activities have important effects on the structure of both the mitochondrial network, and the inner-mitochondrial organization. NLRX1 promotes mitochondrial fission, leading to increased number of mitochondria with reduced numbers of cristae 15.

## MITOCHONDRIAL RESPIRATION AND HUMAN DISORDERS

**Verónica Dumit** (University of Freiburg, Freiburg, Germany) presented her data with emodin, an anthraquinone component of aloe, which selectively affects the proliferation of cancer cells compared to healthy counterparts. Emodin treatment leads to the downregulation of mitochondrial complex I subunits in cancer cells, and triggers mitochondrial fragmentation and ballooning. Emodin shifts isolated yeast mitochondria towards uncoupled respiration, affects the mitochondrial membrane potential, which then leads to impaired import of proteins into the mitochondrial matrix. Yeast cells adapted to fermentation are more vulnerable to emodin treatment than yeast cells with high respiratory capacity. This effect might be comparable to cancer cells, which prefer fermentation in contrast to control cells, which demonstrate higher levels of respiratory activities. In summary, emodin detrimentally affects cells with an inefficient mitochondrial respiratory chain, such as cancer cells.

**Ulrich Brandt** (Radboud University Medical Center, Nijmegen, The Netherlands) presented his work on the structure, function and regulation of the mitochondrial respiratory chain complex I, whose dysfunction is connected to various mitochondrial and mitochondrion-modulated disorders 16. Most notably, he succeeded to resolve the X-ray structure of mitochondrial complex I 17, which will help develop targets for pharmacological treatments to ameliorate disease-associated complex I deficiency and oxidative stress production.

## ROLE OF MITOPHAGY IN HUMAN DISORDERS

**Florian Steinberg** (University of Freiburg, Freiburg, Germany) talked about endocytic Rab GTPases on mitochondrial degradation via mitophagy. Rab GTPases are involved in the regulation of membrane trafficking, including vesicle movement, and membrane fusion. Here, Florian Steinberg presented data that specific Rab GTPases interact with the mitochondrial fission machinery thereby pivotally modulating mitophagy.

**Konstanze Winklhofer** (Ruhr University Bochum, Bochum, Germany) talked about ubiquitin signaling and mitochondrial integrity and their implications for Parkinson’s disease. She introduced into the pivotal role of mitochondrial damage for Parkinson’s disease. Mutations in genes encoding components of the mitophagy machinery, including the E3 ubiquitin ligase Parkin and the protein kinase Pink1, are causative for familiar forms of the human disorder. She challenged the paradigm that the protective role of Pink and Parkin in Parkinson’s disease depends on its role in mitophagy 18,19. Instead, she demonstrated that Parkin requires the NFκB pathway to mediate stress protection, as well as the mitochondrial fusion factor OPA1. Thus, the functions unrelated to mitophagy of Pink1 and Parkin should be further considered in dissecting mechanisms of Parkinson’s disease.

Iron-sulfur cluster biogenesis strictly depends on mitochondrial activity. Many “iron-sulfur diseases” including Friedreich’s ataxia exist, which are caused by mutations in components of the iron-sulfur cluster assembly machinery. **Janina Bergmann** (University of Marburg, Marburg, Germany) presented her work challenging the question of whether mitochondria from iron-sulfur disease patients are removed via Pink1/Parkin-dependent mitophagy. She showed that mitochondria are severely impaired upon deficiency in iron-sulfur cluster biogenesis showing a respiratory insufficiency. Surprisingly, the diseased organelles can maintain a membrane potential via the reverse action of the F_1_/F_O_ ATP synthase using ATP synthesized by increased glycolysis. Consequently, no Pink1/Parkin-dependent mitophagy could be observed confirming that the latter process is strictly dependent on a mitochondrial membrane potential.

## MODELING THE INHERITANCE AND EVOLUTION OF MITOCHONDRIAL DNA

Mutations in mitochondrial DNA (mtDNA) are causative for severe mitochondrial disorders. **Iain Johnston** (University of Birmingham, Birmingham, UK) described work combining mathematical theory and experiments to characterize the evolution of mtDNA within cells, and the inheritance and onset of mitochondrial diseases. He introduced the concept of heteroplasmy, i.e., the fact that one cell may comprise more than one mtDNA haplotype. He demonstrated that the proliferation of one haplotype over another has to be considered in gene therapies to address diseases, and that this phenomenon increases with the genetic distance between the haplotypes 20,21. Damaged mtDNA can be eliminated during mammalian development through a highly debated mechanism called the mtDNA bottleneck; Johnston produced a new, physically motivated, generalizable theoretical model for mtDNA populations during development, allowing the first statistical comparison of proposed bottleneck mechanisms 22.

## ROLE OF MITOCHONDRIA IN WILSON DISEASE AND ALZHEIMER’S DISEASE

**Hans Zischka** (Helmholtz Center Munich, Munich, Germany) demonstrated his work on rat models for Wilson disease, a fatal liver disease. Due to a genetic defect in Wilson disease, copper ions accumulate in liver cells, leading to severe mitochondrial damage. Copper causes mitochondrial structural alterations, and physically impairs the mitochondrial membrane leading to mitochondrial membrane permeabilization and cell death. He demonstrated that treatment of rats with copper chelators reduced the detrimental copper load in mitochondria, rescues mitochondrial function and cell survival, preventing acute liver failure 23.

**Alice Rossi** (University of Padua, Padua, Italy) presented her data describing the effects of Alzheimer’s disease-causing mutations in the protease presenilin 2 affects mitochondrial functionality. She showed that presenilin 2 mutations impaired mitochondrial functionality, resulting in the depletion of cellular ATP levels. She is now elucidating how mutated presenilin 2 linked to familial Alzheimer’s disease induces mitochondrial impairments.

## CONCLUDING REMARKS

The 2^nd^ International Symposium “One mitochondrion, many diseases. Biological and Molecular Perspectives.” in Freiburg (Germany) was the continuation of a previous symposium, which took place in Sherbrooke, Québec, Canada in March 2015. In the first symposium, the role of mitochondria was described in human patients, as well as in mammalian cell culture, yeast, and transgenic mouse models for different diseases, including mitochondrial disorders, inflammatory and neurodegenerative diseases, as well as cancer 24. In the current symposium, the focus was more on basic mitochondrial research, including mitochondrial architecture, structure and integrity, or mitochondrial respiratory chain complexes. We plan our next symposium to take place in Montréal (Québec, Canada) in May 2017. We aim to bring mitochondrial researchers doing basic and clinical research together to discuss diverse human disorders in different model systems.
